# First-Principles Calculations of the Optoelectronic, Vibrational, and Thermoelectric Characteristics of the Novel 2D Nb_3_TeBr_7_ Material

**DOI:** 10.1021/acsomega.6c01767

**Published:** 2026-04-24

**Authors:** Amanda C. Marques, Márcio F. Santos, Willian O. Santos, Elie A. Moujaes, Alexandre C. Dias, Alexandre A. Leitão

**Affiliations:** † Group of Physical Chemistry of Solids and Interfaces, Department of Chemistry, 28113 Federal University of Juiz de Fora, Juiz de Fora 36036-330, MG, Brazil; ‡ Institute of Physics, 28111 Federal University of Bahia, Salvador 40170-115, BA, Brazil; § Institute of Physics and International Center of Physics, 28127 University of Brasília, 70919-970 Brasília, DF, Brazil; ∥ Computational Materials Laboratory, LCCMat, Institute of Physics, University of Brasília, 70919-970 Brasília, DF, Brazil

## Abstract

Solar photovoltaic technology stands at the forefront of the renewable energy revolution, driving the quest for cleaner and more sustainable power sources. Among the countless materials under exploration are two-dimensional (2D) systems—particularly niobium-based monolayers—which have captivated researchers with their remarkable potential. In this work, we harness first-principles calculations to unveil the rich tapestry of electronic, excitonic, optical, transport, and thermoelectric properties exhibited by the Nb_3_TeBr_7_ monolayer. Our computational results confirm the dynamic and mechanical stability of this intriguing material. Through detailed electronic structure analyses using PBE, PBE-SOC, and HSE06 functionals, we uncover a direct HSE06 band gap of 1.56 eV, with electronic states near the Fermi level predominantly arising from Nb atoms. The optical response of the monolayer reveals pronounced anisotropy, as captured by both the Independent Particle Approximation (IPA) and Bethe–Salpeter Equation (BSE) approaches. Estimates of power conversion efficiency (PCE) based on the Shockley–Queisser limit and the spectroscopic-limited maximum efficiency (SLME) indicate PCE values up to 30.40% at the IPA level and 27.40% at the BSE level for the monolayer. For simulated electron doping (via increased Fermi energy) at selected temperatures, the figure of merit (ZT) reaches about 0.1. Simulated hole doping (via lowered Fermi energy) yields ZT values of about 0.05. Although these ZT values are modest, their stability across a range of temperatures suggests that targeted doping with impurities might improve thermoelectric performance.

## Graphical abstract

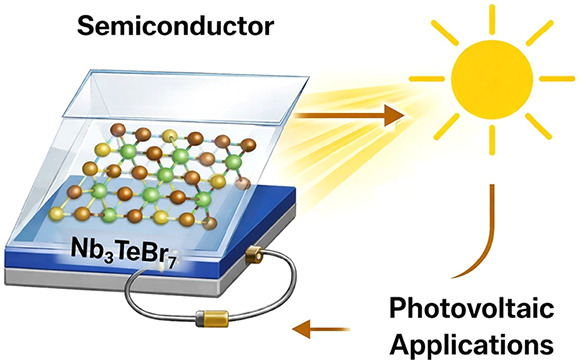

## 1 Introduction

The global transition toward renewable energy has been unequivocally led by the rapid expansion of solar power. By the end of 2023, the world’s installed solar photovoltaic (PV) capacity reached 1418 GW, representing over a third of all renewable capacity.^1^ Projections from the International Energy Agency suggest that renewable’s will constitute 46% of the electricity sector by 2030, with solar and wind power driving nearly all of this growth.^1,2^ As the world confronts the dual challenges of climate change and energy security, PV technology has become an essential pillar for a sustainable future.^3–5^ However, this rapid expansion brings its own set of environmental and logistical challenges tied to current materials. The market is dominated by crystalline silicon solar cells, valued for their high efficiency and durability, yet their energy-intensive manufacturing and the challenge of managing waste from decommissioned panels remain significant concerns.^1,6^ While thin-film technologies like CIGS and CdTe offer alternatives with greater flexibility and lower manufacturing costs, their long-term sustainability is constrained by a reliance on scarce or hazardous elements, such as indium in CIGS and cadmium in CdTe, whose extraction carries a significant ecological footprint.^7^

Addressing these challenges requires a fundamental shift in materials science, a revolution that was catalyzed by the isolation of graphene in 2004.^8–10^ The discovery of monolayer graphene and its fascinating properties has sparked enormous interest in the search for other two-dimensional (2D) materials, leading to the exploration of new families like transition metal dichalcogenides (TMDs)^11–13^ and 2D perovskites.^14–17^ The unique quantum confinement effects in two dimensions gives rise to novel electronic, optical, and mechanical properties fundamentally different from their bulk counterparts, offering unprecedented opportunities to engineer materials for next-generation optoelectronics, spintronics and energy applications.^11,18–20^ For emerging technologies like perovskite^21–23^ and organic solar cells,^24,25^ the most pressing challenges are operational stability and efficiency degradation, as these materials often struggle to withstand long-term exposure to environmental stressors like moisture, oxygen, and thermal cycling.^26^

In the rapidly growing field of 2D materials, niobium-based monolayers have emerged as particularly compelling candidates for advancing photovoltaic technology.^27^ Niobium’s unique electronic structure allows it to significantly enhance the performance of solar materials. For instance, doping perovskites like CsPbBr_3_ with niobium has been shown to greatly improve power conversion efficiency and long-term stability.^28^ The calculated electronic density of states showed that the conduction band is predominantly constructed of doped Nb. These characteristics make them very suitable for solar cells and energy storage applications. The mechanism for such improvements can be seen in other materials like In_2_S_3_, where doping with adequate concentrations of niobium creates a delocalized, occupied intermediate band within the material’s band gap. This feature allows for sub-band gap absorption, leading to higher photocurrent densities compared to the undoped material.^29^ Beyond its role as a dopant, niobium also forms effective electron transport layers (ETLs). For example, depositing niobium pentoxide (Nb_2_O_5_) with a controlled oxygen flow creates conductivity-enhancing oxygen vacancies, which directly boosts solar cell efficiency.^30^

Motivated by the search for a new two-dimensional Niobium-based composite material, the goal of this study is to investigate the structural and optoelectronic properties of the Nb_3_TeBr_7_ monolayer for photovoltaic applications using first principle calculations. This paper is organized as follows: details of the theoretical method are reported Section 2. Results including geometry optimization, cohesive energy, phonon dispersion curves, electronic properties, optical absorption, insights for solar harvesting applications and thermoelectric properties are discussed in Section 3. Finally, a summary is presented in Section 4.

## 2 Theoretical Methodology and Computational Details

Computational modeling was conducted using the Quantum Espresso (QE) package.^31–33^ The exchange-correlation (XC) PBE functional, a generalized gradient approximation (GGA) within the density functional theory (DFT) framework,^34^ was employed alongside scalar-relativistic nonlocal Vanderbilt norm-conserving pseudopotentials from PseudoDojo.^35^ Plane-wave energy cutoffs were set to 80 Ry for the wave function (ecutwfc) and 320 Ry for the charge density (ecutrho). Spin–orbit coupling (SOC) calculations were performed self-consistently using fully relativistic nonlocal Vanderbilt norm-conserving pseudopotentials, maintaining the same energy and charge density cutoffs. In QE, SOC was enabled via lspinorb = .true. and noncolin = .true. For all calculations, a **k**-point mesh of 16 × 16 × 1 and a total energy convergence threshold of 1 × 10^–12^ Ry were applied. The electronic density mixing parameter was fixed at 0.2 using the local Thomas-Fermi (TF) mixing scheme.^36^ Structural optimization utilized the Broyden–Fletcher–Goldfarb–Shanno (BFGS) relaxation algorithm, allowing both atomic positions and in-plane lattice vectors to relax until residual forces were below 1 × 10^–1^ Ry/bohr and stresses below 0.1 kbar. To ensure convergence stability, the maximum number of electronic iterations per cycle was set to 1000. A vacuum spacing of 20 Å was introduced along the nonperiodic direction to eliminate interactions between periodic images. Since PBE underestimates the electronic band gap due to self-interaction errors,^37,38^ the range-separated hybrid exchange–correlation functional HSE06 ^39,40^ (with a range-separation parameter of 0.11 bohr^–1^ and 25% exact exchange) was adopted for a more accurate description of the electronic band gap. The adaptively compressed exchange (ACE) method^41^ was applied with a compressed EXX-operator tolerance of 1 × 10^–8^ Ry to improve computational efficiency, together with the Gygi–Baldereschi scheme for treating the Coulomb divergence.^42^

Phonon dispersion was investigated using a 4 × 4 × 1 phonon grid, which was sufficient to achieve convergence within the framework of Density Functional Perturbation Theory (DFPT).^43^ A stringent convergence threshold of 10^–18^ was applied to the self-consistent solution of the finite displacement equations. Interatomic force constants were obtained using the q2r.x^44^ code. Dynamical matrices computed in reciprocal space were Fourier transformed into real space and subsequently used in the matdyn.x^44^ code to calculate phonon frequencies and dispersion relations along the high-symmetry path in the Brillouin zone.

The elastic constants were evaluated using the ElaStic code^45^ interfaced with QE by computing energy–strain relationships for small in-plane distortions around the relaxed geometry of the freestanding monolayer. The code applies a set of predefined small biaxial compressive and tensile strain patterns. Strain values between −0.8% and 0.8%, in increments of 0.2%, were included. The total energy as a function of strain was fitted to a second-order polynomial using the nine strain points per deformation, from which the second-order elastic constants were extracted. For the present freestanding two-dimensional system, only the independent in-plane elastic constants *C*_11_, *C*_12_, and *C*_66_ were computed. These constants were subsequently used to evaluate the in-plane Young’s modulus and Poisson’s ratio.

Excitonic and optical properties were calculated using both the independent particle approximation (IPA) and the Bethe–Salpeter equation (BSE) levels of theory, with the latter incorporating excitonic quasi-particle effects, utilizing the WanTiBEXOS code.^46^ Electron and hole single-particle states were obtained from a maximally localized Wannier function tight-binding (MLWF-TB) Hamiltonian, constructed directly from HSE06 calculations via the Wannier90 package.^47^ Ten Wannier bands were used to span the relevant valence and conduction manifolds. Both BSE and IPA calculations included the six highest valence bands and the nine lowest conduction bands to describe the linear optical response within the solar emission range (i.e., 0 to 4 eV). A 19 × 19 × 1 **k**-point mesh and a smearing of 0.01 eV were applied for the dielectric functions. The electron–hole Coulomb interaction was modeled using a two-dimensional Coulomb truncated potential (V2DT).^48^

Thermoelectric properties, including the Seebeck coefficient as well as electronic and lattice thermal conductivities, were evaluated at 300, 600, and 900 K using the BoltzWann code^49,50^ interfaced with Quantum Espresso. The electronic band structure was generated using Wannier functions^51,52^ derived from HSE06 ground-state calculations. Electronic lifetimes (τ) for both electrons and holes were assumed to be independent of band and k-point, following a simplified relaxation-time approximation (RTA). Transport properties were computed on an interpolated *k*-grid of 220 × 220 × 1 points. All electronic transport and thermoelectric characteristics reported were obtained using GGA-PBE calculations, employing the same GGA-PBE pseudopotentials and in-plane lattice parameters as those used in the HSE06 geometry optimization.

## 3 Results and Discussion

### 3.1 Structural Properties

The crystal structure of Nb_3_TeBr_7_ monolayer are shown in Figure 1. The structural data utilized in this study were sourced from the Computational 2D Materials Database (C2DB).^53,54^ The primitive cell reveals the arrangement of Nb, Te, and Br atoms, emphasizing the coordination environment and the symmetry of the monolayer.

**Figure 1:**
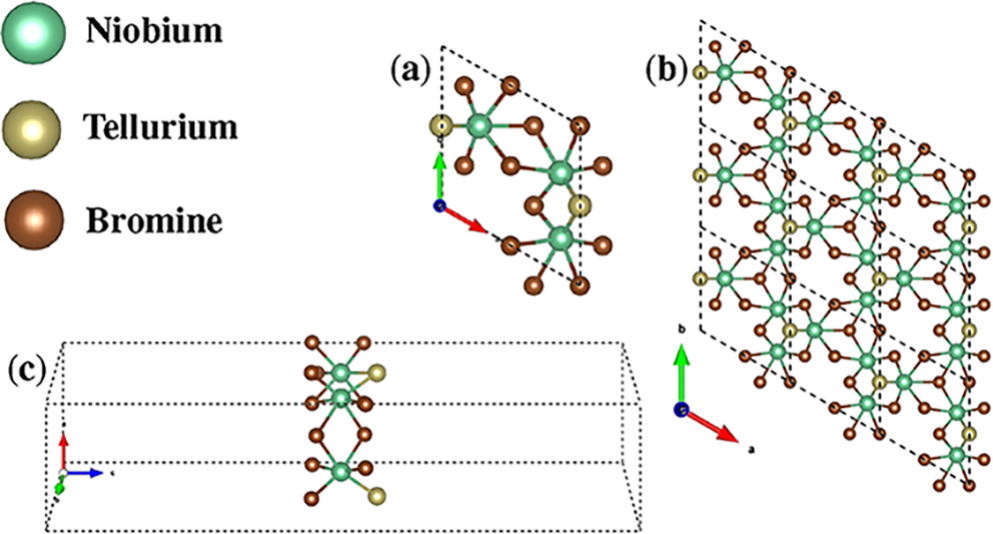
(a) Primitive unit cell of Nb_3_TeBr_7_ and (b) its 3 × 3 × 1 supercell in the *ab* plane. (c) Primitive unit cell of Nb_3_TeBr_7_ in the *ac* plane.

The Nb_3_TeBr_7_ monolayer crystallizes in a hexagonally symmetric Bravais lattice.^55^ The crystal lattice parameters are *a* = *b* = 7.26 Å and *c* = 23.90 Å, with a primitive cell volume of *V* = 465.439 Å^3^. These structural characteristics are consistent with those of other niobium-based monolayers previously reported in the literature.^56–59^

### 3.2 Energetics and Dynamical Stability

Phonon dispersion and cohesive energy calculations are essential in materials research because they yield critical insights into system energetic and dynamical stability. The cohesive energy per atom (*E*_coh/atm_) for the hexagonalstructure of Nb_3_TeBr_7_ are calculated using the following formula^60,61^

1 $$E_{\mathrm{coh}/\mathrm{atm}}=\frac{E_{Nb_{3}\mathrm{TeB}r_{7}}^{\mathrm{tot}}-\left(nE_{\mathrm{Nb}}^{\mathrm{iso}}+mE_{\mathrm{Te}}^{\mathrm{iso}}+pE_{\mathrm{Br}}^{\mathrm{iso}}\right)}{n+m+p}$$

where the “iso” superscript refers to isolated atoms, *E* ^tot^ is the total energy of the structure, and *n*, *m*, and *p* represent the number of Nb, Te, and Br atoms in the unit cell, respectively. The calculated value of *E*_coh/atm_ is −3.07 eV/atom, which confirms the energetic stability of the Nb_3_TeBr_7_ monolayer.

The phonon dispersion of Nb_3_TeBr_7_ monolayer are shown in Figure 2, showing the vibrational frequencies (ω) of the normal modes as a function of the wave vector (**k**) along high-symmetry paths in the first Brillouin zone. The phonon dispersion includes 33 vibrational modes, with three acoustic branches corresponding to the lowest energy bands. These bands show a slight negative value near the Γ point (Figure 2), likely due to numerical inaccuracy in diagonalizing the dynamical matrix. The monolayer is considered dynamically stable, as there are no significant negative frequencies or soft phonon modes along the high-symmetry path in the Brillouin zone.

**Figure 2:**
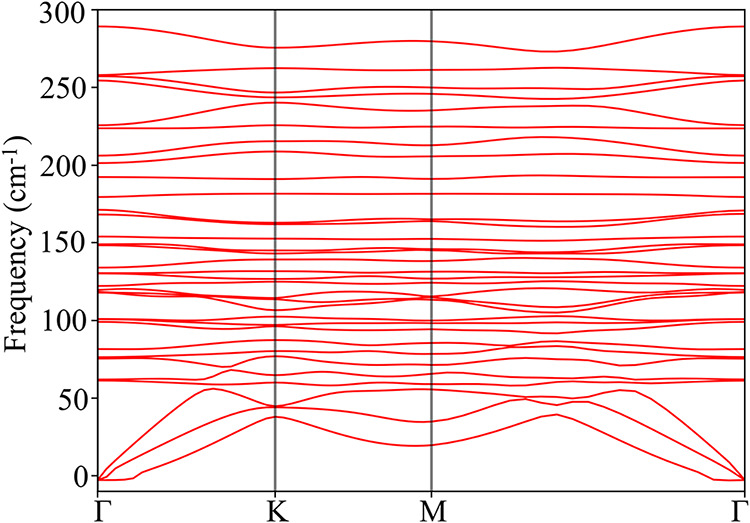
Phonon dispersion of the Nb_3_TeBr_7_ monolayer along the Γ-K-M-Γ path calculated at PBE level.

### 3.3 Mechanical Stability

The mechanical stability of crystalline materials is determined by elastic constants, which quantify the response to applied stress and must satisfy thermodynamic criteria based on the positive definiteness of the elastic stiffness matrix. In a two-dimensional hexagonal structure, there are three independent in-plane elastic constants: C_11_, C_22_, and C_12_. For pure isotropic materials, C_11_ equals C_22_. The dependent elastic constant C_66_ is defined as (C_11_–C_12_)/2. C_11_ and C_22_ measure the response along the *x* and *y* directions, respectively, while C_12_ describes the response along the *y* direction when stress is applied along the *x* direction. C_66_ represents the shear modulus of the monolayer.

The above constants allow calculation of the Poisson ratio (ν_2D_), which indicates the extent of expansion or contraction perpendicular to the applied stress. Mathematically, ν_2D_ is given by C_12_/C_11_ and C_12_/C_22_ along the *x* and *y* directions, respectively. Additionally, the 2D Young’s modulii (*Y*_2D_) along the *x* and *y* directions can be obtained via the expressions

2 $$\begin{array}{l} Y_{2D}^{x}=\frac{C_{11}C_{22}-C_{12}^{2}}{C_{11}}\\ Y_{2D}^{y}=\frac{C_{11}C_{22}-C_{12}^{2}}{C_{22}} \end{array}$$

The Born criteria for mechanical stability require that C_11_ > 0, C_22_ > 0, C_66_ > 0, C_11_ > C_12_, and ν_2D_ < 0.5.^62–65^ Our calculations yield C_11_ = 62.74 N/m, C_22_ = 61.59 N/m, C_12_ = 9.60 N/m, and C_66_ = 15.29 N/m, indicating slight anisotropy (∼0.5%) along the *x* and *y* directions. This anisotropy is also evident in the Poisson ratios (ν_2D_^*x*^ = 0.153, ν_2D_^*y*^ = 0.156) and Young’s moduli (*Y*_2D_^*x*^ = 60.12 N/m, *Y*_2D_^*y*^ = 61.24 N/m). This difference is attributed to minor atomic displacements that occurred following structural optimization, which are considered acceptable density functional theory (DFT) noise. Since all diagonal elastic constants are positive and the Poisson ratios are below 0.5, we conclude that the Nb_3_TeBr_7_ monolayer is mechanically stable.

### 3.4 Electronic Properties

The electronic band structure and density of states of Nb_3_TeBr_7_ monolayer are shown in Figure 3, with the Fermi level set at the valence band maximum. The band structure calculated using PBE (blue curves) and PBE-SOC (black dashed curves) are nearly identical along the entire high-symmetry path. The inclusion of SOC introduces slight band separations and smooths certain dispersions, particularly above 2 eV and just below the Fermi level. These minor shifts suggest that SOC predominantly influences the more dispersive states associated with Nb and Te.

**Figure 3:**
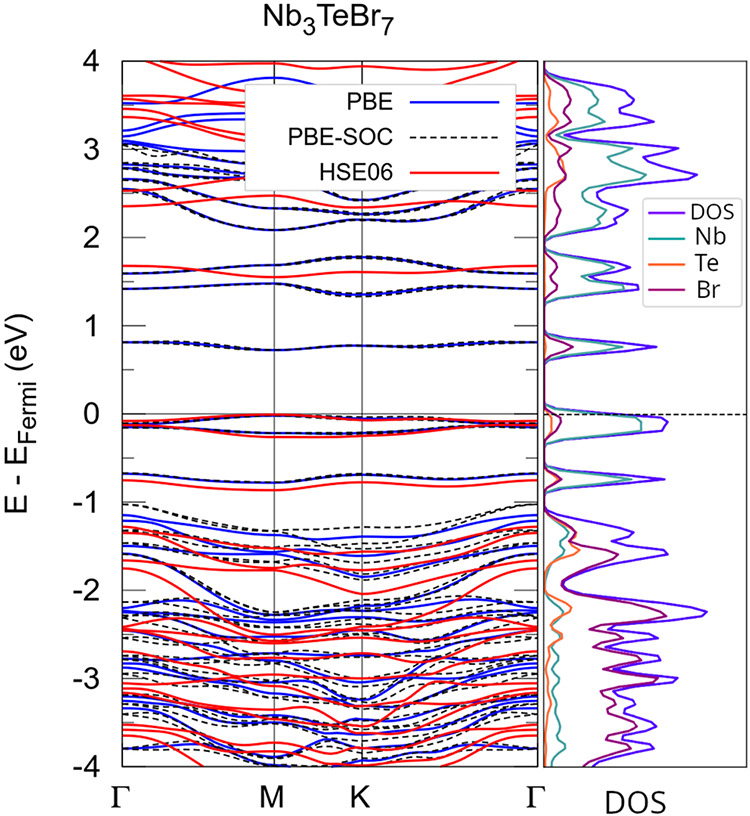
Band structures of 2D Nb_3_TeBr_7_ monolayer obtained at the PBE, PBE+SOC, and the HSE06 levels of functionals. The projected density of states (pDOS), obtained from PBE functionals, is also shown. The Fermi level is set at 0 eV.

The total density of states (DOS) and projected density of states (pDOS) were calculated using the PBE functional and are shown in Figure 3. The pDOS indicates higher electron density at Nb atoms near the Fermi level, with some contribution from Br atoms, Te orbitals contribute only to deeper valence and conduction bands. The partially filled *d*-orbitals of Nb atoms frequently cross the Fermi level. Nb *d*-orbitals and Br *p*-orbitals dominate the electronic structure just above the Fermi level, while Te orbitals contribute mainly to deeper regions of the valence and conduction bands.

As expected, the PBE functional underestimates the band gap, using HSE06 functional the electronic band gap goes from 0.68 eV at PBE­(PBE-SOC) to 1.56 eV at HSE06, being direct in both cases, classifying Nb_3_TeBr_7_ as a direct band gap semiconductor. As SOC effects are very weaken in the vicinity of Fermi level, as shown in the electronic band structure, SOC effects are neglect in HSE06 electronic properties and in the next calculations.

### 3.5 Excitonic and Optical Properties

From the exciton band structure shown in Figure 4, we observe that this monolayer presents a direct exciton ground state (at Γ) of 0.89 eV. Comparing the fundamental band gap and exciton ground state we can estimate an exciton binding energy of 676 meV which is in the expected region for 2D materials,^66–68^ that tends to be higher due the quantum confinement.

**Figure 4:**
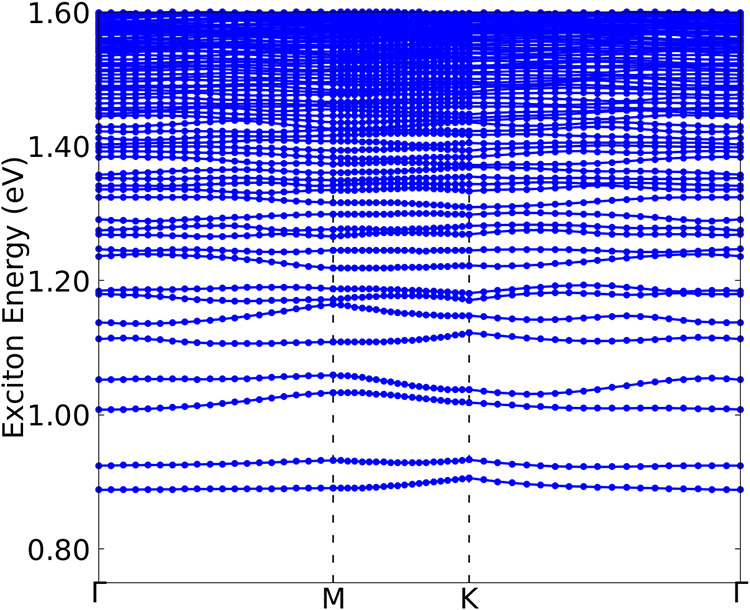
(colon online) Exciton band structure of the Nb_3_TeBr_7_ monolayer calculated using the BSE method.

Figure 5 presents the linear optical response, including absorption coefficient, refractive index, and reflectivity, for BSE (solid curves) and IPA (dashed curves) with incident linear light polarized along *x̂* (blue) and *ŷ* (red). The absorption coefficient (Figure 5(a)) shows strong optical anisotropy, with a more intense response for light polarized along *ŷ* at both IPA and BSE levels. This suggests that electronic transitions are stronger when the electric field is aligned with *ŷ*, likely due to greater orbital overlap or a higher density of states in this direction. As expected, excitonic quasi-particle effects cause a red shift in the optical band gap from 1.56 to 0.89 eV. Despite the anisotropy, the optical band gap remains the same for both polarizations. The monolayer absorbs in the infrared, visible, and UV regions at both BSE and IPA levels, although at IPA it absorbs only a small fraction of the infrared region due to the direct band gap of 1.56 eV. Absorption is most intense in the UV region. Figure 4 shows a direct exciton ground state at Γ with an energy of 0.89 eV.

**Figure 5:**
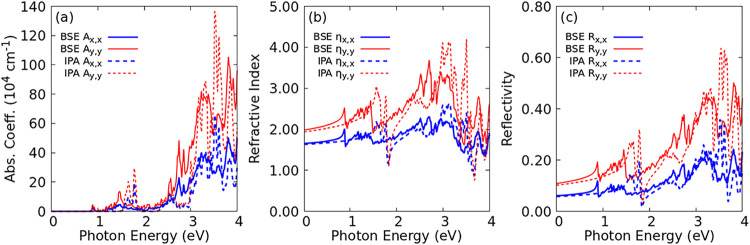
(a) Absorption coefficient at BSE (solid lines) and IPA (dashed lines) levels, for linearly polarized light along the *x*-axis (red curves) and *y*-axis (blue curves). (b) Refractive index at BSE (solid lines) and IPA (dashed lines) levels, for linearly polarized light along the *x*-axis (red curves) and *y*-axis (blue curves). (c) Reflectivity at BSE (solid lines) and IPA (dashed lines) levels, for linearly polarized light along the *x*-axis (red curves) and *y*-axis (blue curves).

The refractive index (Figure 5(b)) and reflectivity (Figure 5(c)) both show higher values in the *ŷ* direction across the solar emission region (0 to 4 eV). BSE and IPA results are similar, with the main difference being a redshift of some sharpened peaks in the infrared and visible regions due to quasi-particle effects. For *x̂* incident light, the refractive index ranges from 1.64 to 2.10, while for *ŷ* it ranges from 1.0 to 4.0. At the BSE level, reflectivity shows a local maximum near 1.5 eV, reaching approximately 10% for *x̂* and 40% for *ŷ*. At the IPA level, this peak is located around 2.0 eV. Maximum reflectivity occurs in the UV region near 3.5 eV, exceeding 60% for *ŷ* and approaching 35% for *x̂*.

The linear optical response, when considering excitonic effects, is highly sensitive to the external dielectric environment.^66^ The same applies to the exciton binding energy, as the external dielectric environment can weaken the electron–hole Coulomb interaction. BSE calculations were performed for a monolayer in vacuum, where quasi-particle effects are strongest, representing the upper limit for exciton binding energy. In contrast, IPA calculations neglect these effects. Experimentally, the actual optical response is expected to fall between the BSE and IPA results.

### 3.6 Insights in Solar Harvesting Efficiency

The solar harvesting efficiency of Nb_3_TeBr_7_ monolayer was evaluated using power conversion efficiency (PCE), calculated by both the Shockley–Queisser limit (SQ-limit)^69,70^ and the spectroscopy limited maximum efficiency (SLME)^71^ methods. The mathematical formalism for both approaches is detailed in the WanTiBEXOS paper.^46^ The SQ-limit requires only the optical band gap, while the SLME also incorporates the absorption coefficient and a recombination fraction, determined by the difference between direct and indirect electronic band gaps at the IPA level and between direct and indirect excitonic ground states at the BSE level. PCE was estimated for a solar cell at 300.0 K using the AM1.5G solar emission spectrum model.^72^ For SLME absorbance, we considered a monolayer effective thickness of 6.41 Å, calculated as the sum of the monolayer thickness (3.11 Å) and a van der Waals length (3.30 Å), following Bernardi et al.^73^ to provide a realistic optical absorbance description. Absorbance was calculated from the total absorption spectrum,^68,74–76^ defined as the sum of the *x̂* and *ŷ* components.

At the SQ limit, we obtained a PCE^SQ^ of 30.41% at the IPA level and 27.40% at the BSE level. This difference is due to the optical band gap redshift caused by excitonic effects. As the optical band gap moves further from 1.33 eV, the PCE^SQ^ increases. At the SLME level, we obtained a PCE^SLME^ of 0.60% at IPA and 0.40% at BSE. The large difference between the SQ-limit and SLME results is explained by the monolayer’s small thickness, which leads to a low incident photon absorbance rate. The SQ-limit assumes all incident photons with energy equal to or greater than the optical band gap are absorbed, which does not occur in this material due to its atomic thickness.

Jariwala et al.^77^ proposed the use of light trapping techniques to increase monolayer absorbance of incident photons with energies equal to or greater than the optical band gap, approaching 100%. In this study, the power conversion efficiency (PCE) was estimated under these conditions (PCE_max_^SLME^), treating the absorbance curve as a Heaviside function equivalent to the Shockley–Queisser limit, while also accounting for the recombination fraction. This consideration reflects the difference between direct and indirect electronic band gaps in the IPA case and between indirect and direct excitonic ground states in the BSE case. Under these assumptions, a PCE_max_^SLME^ of 30.40% was obtained for IPA and 27.40% for BSE, which are the same values of the PCE^SQ^, being justified by the direct band gap and direct excitonic ground state of this material.

Regardless of the method used to estimate the power conversion efficiency (PCE), the present results represent the upper limit for the solar harvesting efficiency of this monolayer. Achieving efficiencies closer to these values requires addressing additional challenges in solar cell architecture, such as identifying suitable electron and hole transport layers, which may require years of research. Because excitonic effects are highly sensitive to the external dielectric environment, and the monolayer will be positioned between electron and hole transport layers in a solar cell, these effects are expected to be weaker than those observed in the current results, which assume the monolayer is in vacuum. Consequently, a PCE value between the IPA and BSE results is likely to be more realistic.

Despite these higher values of PCE^SQ^ and PCE_max_^SLME^, both assume 100% photon absorbance, which is not realistic given the atomic monolayer thickness. Addressing this limitation through light trapping techniques presents significant engineering challenges, may introduce additional architectural issues in the solar device, or could require energy inputs that reduce the commercial attractiveness of the technology.

### 3.7 Electronic Transport

The determination of electronic carrier mobilities for electrons and holes is essential in semiconductor physics and materials science, as these values quantify the rate at which charge carriers drift under an electric field and directly influence device efficiency and performance.^78,79^ Electron and hole mobilities govern the operational speed and power efficiency of transistors, diodes, and integrated circuits. High carrier mobilities facilitate faster switching speeds and reduced energy dissipation in field-effect transistors (FETs),^80,81^ which is critical for high-frequency electronics and advanced computing applications.

Assuming that acoustic phonons dominate over optical phonons, a central premise of deformation potential theory (DPT),^82^ a two-dimensional version of the empirical expression proposed by Bardeen and Shockley is employed^63,83–86^

$$\mu_{i,j}^{2D}=\frac{2e\hbar^{3}C_{\mathrm{jj}}}{3k_{B}T|m_{i,j}^{*}{|}^{2}E_{1i}^{2}}$$

*e* denotes the electron charge, ℏ represents the reduced Planck constant, *k*_B_ is Boltzmann’s constant, *T* indicates temperature, and m* denotes the effective mass. The subscripts *i* and *j* specify the charge carrier and direction, respectively. C_*jj*_ refers to the elastic constants in direction *j* as calculated previously, and *E*_1*i*_ is the deformation potential of charge carrier *i*, which can be calculated as follows

$$E_{1i}=\frac{\partial E_{\mathrm{edge},i}}{\partial \delta }$$

where *E*_edge,*i*_ represents the energy of the valence band maximum (VBM) for holes and the conduction band minimum (CBM) for electrons, respectively, and δ is the strain strength applied to the unit cell. Within the constant time approximation (CTA), which assumes the dominance of the acoustic phonons, the electronic lifetime (τ*_e_*) is determined by mobility and effective mass as follows

$$\tau_{i,j}=\frac{\mu_{i,j}m_{i,j}^{*}}{e}$$

Since both the mobility (μ) and effective mass (m*) depend on the carrier type and direction, the relaxation time (τ) will also depend on these two quantities.

Figure 6 shows electron and hole mobilities and lifetimes as a function of temperature along the *x* and *y* directions. Electron mobilities (lifetimes) exceed hole mobilities (lifetimes) in both directions, and mobilities (lifetimes) are higher along the *y* direction for both carriers. Table 1 displays the exact values of μ and τ at 300, 600, and 900 K along the *x* and *y* directions. m* and *E*_1_ are also included. Although the Nb_3_TeBr_7_ monolayer has low electron and hole mobility values, it is well suited for use in disordered environments such as organic photodetectors, gas sensors, and photovoltaics. It is also a strong candidate for flexible electronics rather than fast transistors.

**Table 1:** Effective Mass (m*), Deformation Potential (E_1_), Electronic Mobility (μ), and Electronic Lifetime (τ) of Electrons and Holes along the *x* and *y* Directions at 300, 600, and 900 K for the 2D Nb_3_TeBr_7_ Structure^a^

direction	temperature (K)	charge carrier	m*	*E*_1_ (eV)	μ (cm^2^/(V.s))	τ (fs)
*x*	300	electron	2.03*m*_*e*_	–3.77	15.25	17.60
		hole	–5.19*m*_*e*_	–4.15	1.92	5.69
	600	electron	2.03*m*_*e*_	–3.77	7.63	8.80
		hole	–5.19*m*_*e*_	–4.15	0.96	2.84
	900	electron	2.03m_*e*_	–3.77	5.08	5.87
		hole	–5.19*m*_*e*_	–4.15	0.64	1.89
*y*	300	electron	1.88*m*_*e*_	–3.65	18.74	20.00
		hole	–2.86*m*_*e*_	–4.38	5.57	9.07
	600	electron	1.88*m*_*e*_	–3.65	9.37	10.00
		hole	–2.86*m*_*e*_	–4.38	2.78	4.53
	900	electron	1.88m_*e*_	–3.65	6.25	6.67
		hole	–2.86*m*_e_	–4.38	1.85	3.02

a *m_e_* is the free electron’s mass.

**Figure 6:**
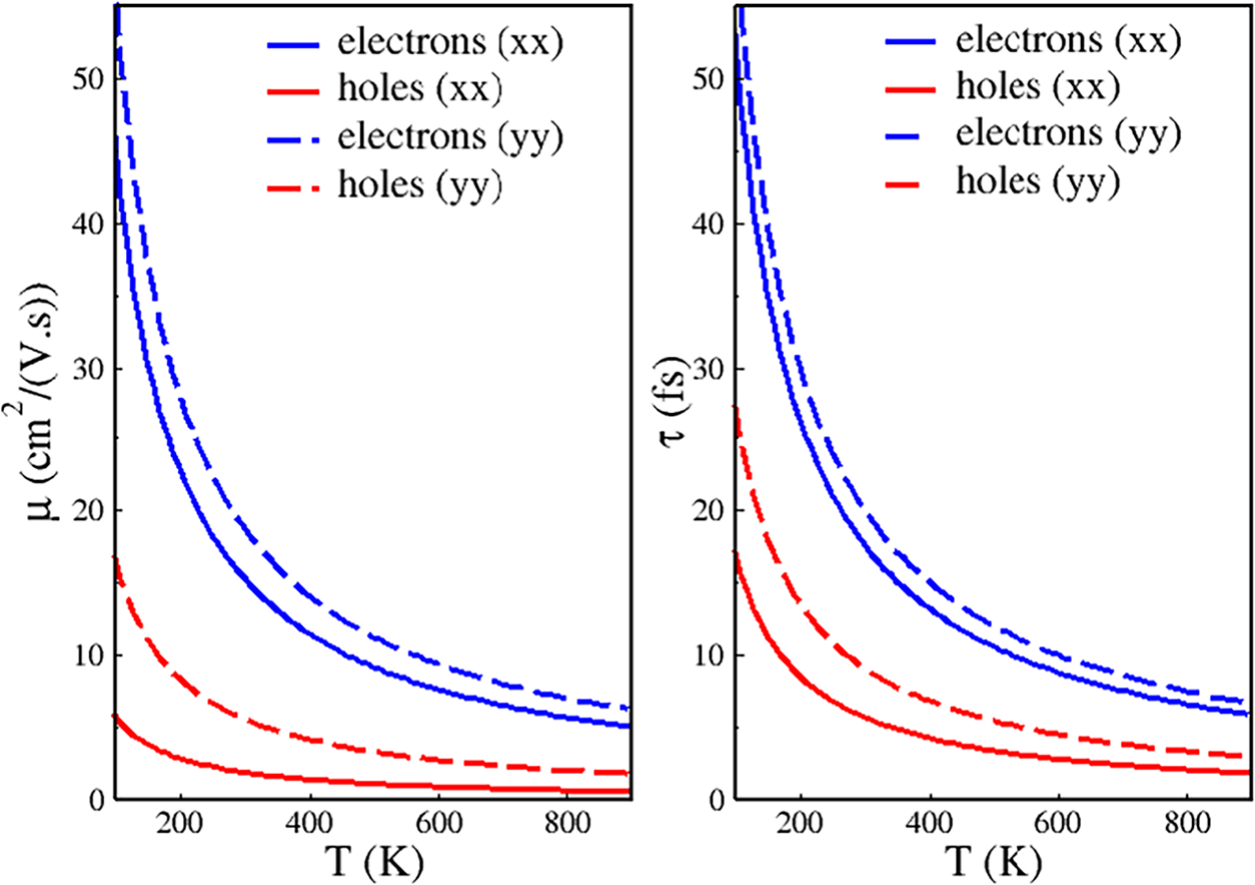
Electron and hole mobilities (μ) and lifetimes (τ) along the *x* and *y* directions as a function of temperature T.

Despite the lower electron and hole mobility of Nb_3_TeBr_7_ monolayer shown in Table 1, our monolayer works as a photoabsorber in the solar device, where the photoexcited electron–hole pair, after exciton lifetime, was dissociated in single electron and hole particles, being transported by the electron and hole transport layers (ETL and HTL), which sandwiches the Nb_3_TeBr_7_ monolayer photoabsorber, these ETL and HTL should have a good electron and hole mobility to achieve the PCE values previously reported.

### 3.8 Thermoelectric Properties

The thermoelectric properties of the Nb_3_TeBr_7_ monolayer are evaluated using Boltzmann transport theory^87,88^ and Wannier functions.^51,52^ The Seebeck coefficient (*S*), electronic conductivity (σ), and electronic thermal conductivity (κ_*e*_) are defined by the following equations^62,63,65,84–86^

$$\begin{array}{l} \kappa_{e}^{\alpha \beta }\left(E\right)=\frac{1}{\mathrm{NVT}}\sum_{n,k}\tau_{n,k}v_{g}^{\alpha }\left(n,k\right)v_{g}^{\beta }\left(n,k\right){\left(E_{n,k}-\eta \right)}^{2}\times \left[-\frac{\partial f\left(T,E,\eta \right)}{\partial E}\right]\\ \sigma^{\alpha \beta }\left(T,\eta \right)=\frac{e^{2}}{\mathrm{NV}}\int \sum_{n,k}\tau_{n,k}v_{g}^{\alpha }\left(n,k\right)v_{g}^{\beta }\left(n,k\right)\times \delta \left(E-E_{n,k}\right)\left[-\frac{\partial f\left(T,E,\eta \right)}{\partial E}\right]dE\\ S^{\alpha \beta }\left(T,\eta \right)=\frac{1}{\mathrm{eVT}\sigma^{\alpha \beta }\left(T,\eta \right)}\int \kappa_{e}^{\alpha \beta }\left(E-\eta \right)\times \left[-\frac{\partial f\left(T,E,\eta \right)}{\partial E}\right]dE \end{array}$$

Here, α and β denote the tensor components, *N* represents the electronic **k** points sampled in the Brillouin zone, *V* is the unit cell volume, and η denotes the chemical potential. *E*_*n*,*k*_ and τ_*n*,*k*_ correspond to the energy and electronic lifetime of the *n* ^th^ band at the *k* ^th^ point, respectively. The term *v*_*g*_^α^(*n, k*) refers to the α^th^ component of the electronic group velocity *v*_*g*_.

The above equations were solved using the relaxation-time approximation (RTA) and the rigid-band approximation (RBA). In the RTA, a single value for the electronic lifetime (τ) was employed, rather than τ_*n*,*k*_. As a result, τ is factored out of the summation and integration operations in the relevant expressions. The values provided to the BoltzWann code are those listed in Table 1. The inclusion of η in these expressions allows for manual adjustment of the Fermi level (*E*_F_) to simulate electron and hole doping. Under the RBA,^89,90^ it is assumed that the electronic band structure remains largely unchanged with moderate shifts in *E*_F_, which is a reasonable assumption for light doping, where η does not deviate significantly from *E*_F_. In our calculations, we will show results for −0.3 eV ≤ η – *E*_F_ ≤ 0.3 eV.

To ensure vacuum-independent results, a simple correction is applied to σ and κ_*e*_. Specifically, the calculated values are multiplied by a factor *V*/*V*′,^63^ where *V* represents the volume including the manually added vacuum distance (*z*_vac_), and *V*′ denotes the volume corresponding solely to the material thickness (*t*). Mathematically, *V* = *A* × (*t* + *z*_vac_) and *V*′ = *A* × *t*, where *A* is the area of the unit cell. Thus, *V*/*V*′ simplifies to (*t* + *z*_vac_)/*t*.

From the above equations, we can define the figure of merit (zT) as

$$\mathrm{zT}=\frac{S^{2}\sigma T}{\kappa_{e}+\kappa_{L}}$$

where κ*_L_* is the lattice thermal conductivity. The term *S* ^2^σ in the numerator is called the power factor (PF). In thermoelectric devices, it quantifies a material’s capacity to convert a temperature gradient into electrical power via its electronic transport properties. Higher power factor results in higher output power for these devices. On the other hand, zT quantifies the thermoelectric performance and efficiency of materials. Values of zT greater than 1 are generally regarded as suitable for practical thermoelectric applications, while values above 1.5 to 2 are considered excellent or record-setting.

To determine zT, we still need to know how to evaluate κ*_L_*. Generally speaking, it is determined via the expression

$$\kappa_{L}^{\alpha \beta }=\frac{1}{\mathrm{NV}}\sum_{\mathrm{q⃗},s}c_{v}\left(\mathrm{q⃗},s\right)v_{g}^{\alpha }\left(\mathrm{q⃗},s\right)v_{g}^{\beta }\left(\mathrm{q⃗},s\right)\tau_{\mathrm{ph}}\left(\mathrm{q⃗},s\right)$$

*c*_*v*_ denotes the phonon specific heat, and τ_ph_ represents the phonon lifetime, both associated with the phonon wave-vector *q⃗* in the phonon dispersion branch *s*, respectively.

Computing κ*_L_* using density functional theory (DFT) is computationally intensive because it requires determining third-order anharmonic coefficients, even for systems with small unit cells. For systems with large unit cells, such as the one considered in this study, the evaluation is even more complex. This challenge can be addressed by employing the Slack model,^91–93^ which replaces the original definition with an empirical expression

$$\kappa_{L}^{\mathrm{slack}}=A\frac{\mathrm{M̅}V^{1/3}\theta_{D}^{3}}{\gamma^{2}\mathrm{nT}}$$

where *M̅* is the average atomic mass, *V* is the volume, *n* is the number of atoms in the unit cell, and θ_D_ and *T* are the traditional Debye temperature^94^ and the usual temperature, respectively. γ is the Grüneisen parameter^95^ and *A* is a constant adjusted in the Slack model and is given in terms of γ given by^96,97^

$$A=\frac{2.4\times 10^{-6}}{1-\frac{0.514}{\gamma }+\frac{0.228}{\gamma^{2}}}$$

with

$$\gamma =\frac{3}{2}\left(\frac{1+\nu }{2-3\nu }\right)$$

ν being the Poisson ratio along the *x* or *y* directions.^63,85^ By adopting this model, we suppose that the acoustical phonon modes play the dominant role in the heat transport process. Consistent with the method applied to σ and κ_*e*_, κ_*L*_^Slack^ is rescaled by multiplying the expression by (*t*/(*t* + *z*_vac_))^1/3^, since *V* appears in the numerator raised to the one-third power. Because the Slack model omits third-order anharmonic terms, the predicted values of κ*_L_* are likely overestimated relative to density functional theory (DFT) results. DFT typically accounts for detailed mode-resolved scattering, leading to shorter phonon lifetimes and, consequently, lower κ*_L_* values. Therefore, the resulting zT values are underestimated and represent lower bound estimates.

Figure 7 shows vacuum-independent PBE-based thermoelectric properties along the *x* and *y* directions at 300, 600, and 900 K. The data reflect a maximum electron or hole doping of 0.3 eV above or below *E*_F_, as previously described. Quantities such as σ and κ_*e*_ (and thus PF and zT) are anisotropic, with distinct values in each direction. Similar to the mobility and the electronic lifetime, κ_*L*_ decreases as temperature increases.

**Figure 7:**
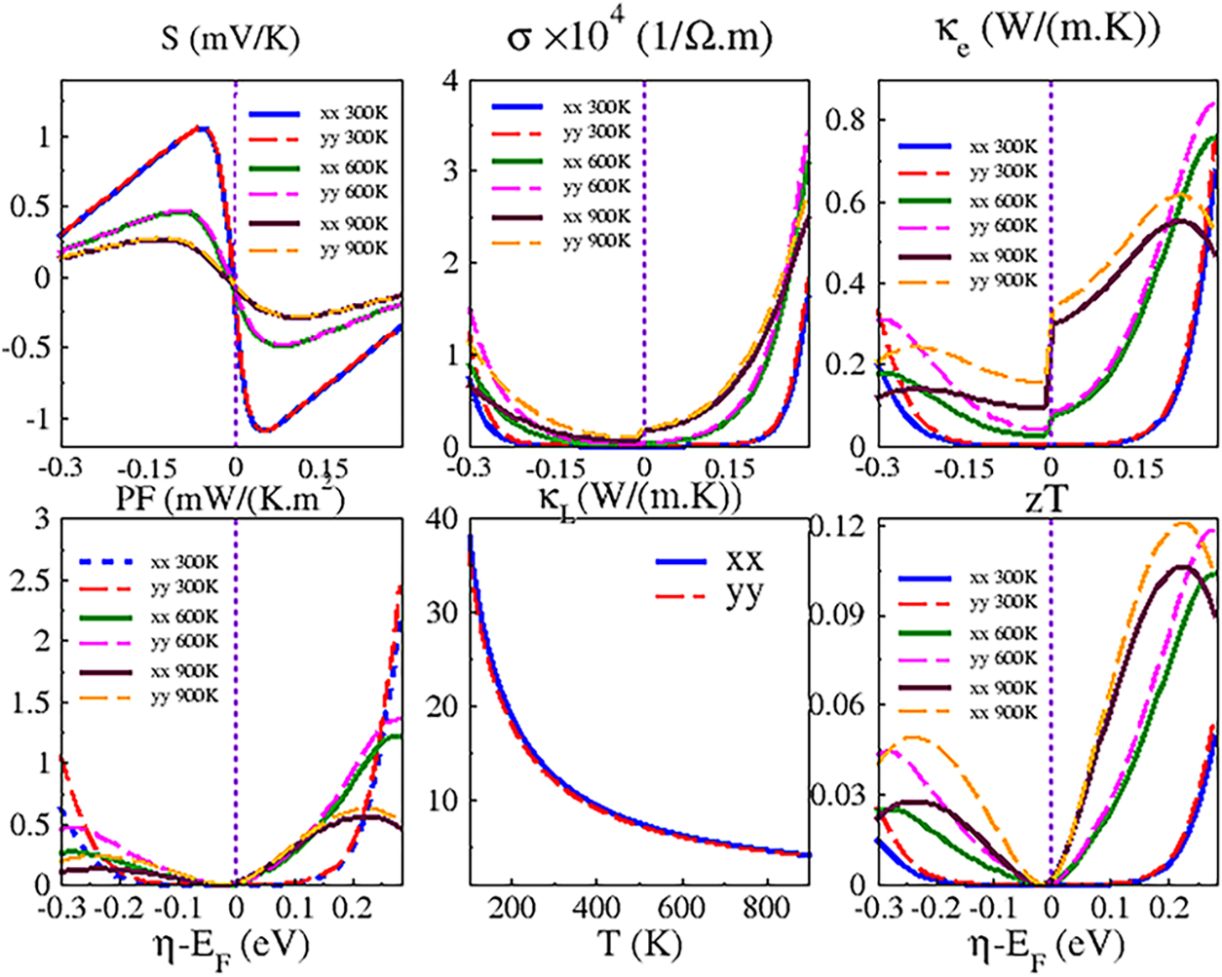
Seebeck coefficient (*S*), electronic conductivity (σ), thermal electronic conductivity (κ_*e*_), thermal lattice conductivity (κ_*L*_), power factor (*S* ^2^σ), and figure of merit (zT) of the Nb_3_TeBr_7_ monolayer along the *x* and *y* directions at 300, 600, and 900 K.

For the undoped case, specifically when η equals *E*_F_ and for comparative purposes, the results are summarized in Table 2. The Seebeck coefficients are negative, indicating that electrons serve as the primary charge carriers. These coefficients decrease in absolute value as temperature increases and exhibit isotropy for both electrons and holes. Both σ and κ_*e*_ increase with temperature and display higher values for holes. The zT values remain low and are marginally higher for electrons.

**Table 2:** Values of *S*, *σ*, *κ*_*e*_, *κ_L_*, and zT of the Nb_3_TeBr_7_ Monolayer along the *x* and *y* Directions at 300, 600, and 900 K Falong the *x* or *y* Directions or the Undoped Case, That is When *η* = *E*_F_

direction	temperature (K)	*S* (mV/K)	σ (1/(Ω.m))	κ_*e*_ (W/(m.K))	κ_*L*_ (W/(m.K))	zT
*x*	300	–0.27	0.55	2.77 × 10^–4^	12.64	9.54 × 10^–7^
	600	–0.14	276.64	0.074	6.32	4.97 × 10^–4^
	900	–0.083	1624.25	0.29	4.21	2.24 × 10^–3^
*y*	300	–0.24	0.63	3.18 × 10^–4^	12.09	8.87 × 10^–7^
	600	–0.12	317.48	0.085	6.05	4.12 × 10^–4^
	900	–0.065	1869.25	0.35	4.03	1.63 × 10^–3^

Doping represents a promising approach to enhance the thermoelectric efficiency of this material. Within the investigated η – *E*_F_ range, the highest zT values are 0.06 (0.07), 0.10 (0.12), and 0.11 (0.12) at 300, 600, and 900 K along the *x* (*y*) direction, respectively. The optimal values at 300 and 600 K occur at η – *E*_F_ = 0.3 eV, shifting to η – *E*_F_ = 0.22 eV at 900 K. In contrast, hole doping yields lower maxima of 0.015 (0.025) at η – *E*_F_ = −0.3 eV, 0.025 (0.044) at η – *E*_*F*_ = −0.28 eV, and 0.028 (0.05) at η – *E*_F_ = −0.24 eV for 300, 600, and 900 K along the x (y) direction, respectively. These results indicate that electron doping may enable the development of more efficient thermoelectric devices.

## 4 Conclusion

We investigate the electronic, excitonic, and thermoelectric properties of the Nb_3_TeBr_7_ monolayer. Its negative cohesive energy and lack of imaginary frequencies in the phonon dispersion confirm energetic and dynamical stability. The monolayer also meets the Born criteria for mechanical stability, with slight anisotropy observed along the *x* and *y* directions.

The electronic properties indicate that the material is a semiconductor with an HSE06 direct band gap of 1.56 eV, and spin–orbit coupling is negligible. The projected density of states demonstrates that the primary contributions near the Fermi level originate from Nb electrons.

Excitonic effects are notable in this monolayer, with an exciton binding energy of 676 meV and a direct excitonic ground state at 0.89 eV. The monolayer also shows appreciable optical anisotropy, both at the Bethe-Salpeter equation (BSE) and independent-particle approximation (IPA) levels, and absorbs light across the infrared, visible, and ultraviolet ranges. For solar-energy applications, the Nb_3_TeBr_7_ monolayer exhibits promising photovoltaic performance, yielding a maximum power-conversion efficiency of 30.40% at the IPA level and 27.40% at the BSE level, under the idealized assumption that the solar-cell architecture can harvest all incident photons.

Upon manually raising and lowering the Fermi energy, the material exhibits a maximum figure of merit of 0.1 for electron doping and 0.05 for hole doping at 900 K, indicating a preference for electron doping. By adding impurity atoms to dope the material, we could improve the values of zT. Thus, the Nb_3_TeBr_7_ monolayer holds potential for future thermoelectronic devices.

## References

[1] Ali A. O., Elgohr A. T., El-Mahdy M. H., Zohir H. M., Emam A. Z., Mostafa M. G., Al-Razgan M., Kasem H. M., Elhadidy M. S. (2025). Advancements
in photovoltaic technology: A comprehensive review of recent advances
and future prospects. Energy Convers. Manage.:X.

[2] Barbosa L. S., Santos W. O., Costa F. S., Moreira E., Azevedo D. L. (2026). Nb2CBr2MXene
Monolayer as a Novel Material: A First Principle Study. Adv. Theory Simul..

[3] Jaiswal K. K., Chowdhury C. R., Yadav D., Verma R., Dutta S., Jaiswal K. S., Sangmesh B., Karuppasamy K. S. K. (2022). Renewable
and Sustainable Clean Energy Development and Impact on social, economic,
and Environmental Health. Energy Nexus.

[4] Singh B. P., Goyal S. K., Kumar P. (2021). Solar PV cell
materials and technologies:
Analyzing the recent developments. Mater. Today:
Proc..

[5] Maka A. O. M., Alabid J. M. (2022). Solar Energy
Technology and Its Roles in Sustainable
Development. Clean Energy.

[6] Owusu P. A., Sarkodie S. A. (2016). A Review of Renewable
Energy sources, Sustainability
Issues and Climate Change Mitigation. Cogent
Eng..

[7] Lee T. D., Ebong A. U. (2017). A review of thin film solar cell technologies and challenges. Renewable Sustainable Energy Rev..

[8] Novoselov, K. S. ; Geim, A. K. ; Morozov, S. V. ; Dubonos, S. V. ; Zhang, Y. ; Jiang, D. Room-temperature electric field effect and carrier-type inversion in graphene films. 2004 arXiv:cond-mat/0410631 arXiv.org e-Printarchive https://arxiv.org/abs/cond-mat/0410631.

[9] Novoselov K. S., Geim A. K., Morozov S. V., Jiang D., Katsnelson M. I., Grigorieva I. V., Dubonos S. V., Firsov A. A. (2005). Two-dimensional
gas of massless Dirac fermions in graphene. Nature.

[10] Novoselov K. S., Jiang Z., Zhang Y., Morozov S. V., Stormer H. L., Zeitler U., Maan J. C., Boebinger G. S., Kim P., Geim A. K. (2007). Room-Temperature Quantum Hall Effect in Graphene. Science.

[11] Ahn E. C. (2020). 2D materials
for spintronic devices. npj 2D Mater. Appl..

[12] Aftab S., Iqbal M. Z., Haider Z., Iqbal M. W., Nazir G., Shehzad M. A. (2022). Bulk Photovoltaic Effect in 2D Materials for Solar-Power
Harvesting. Adv. Opt. Mater..

[13] Neilson K. M., Hamtaei S., Nazif K. N., Carr J. M., Rahimisheikh S., Nitta F. U., Brammertz G., Blackburn J. L., Hadermann J., Saraswat K. C., Reid O. G., Vermang B., Daus A., Pop E. (2024). Toward Mass Production
of Transition
Metal Dichalcogenide Solar Cells: Scalable Growth of Photovoltaic-Grade
Multilayer WSe_2_ by Tungsten Selenization. ACS Nano.

[14] Aftab S., Iqbal M. Z., Hussain S., Hegazy H. H., Saeed M. A. (2023). Transition
metal dichalcogenides solar cells and integration with perovskites. Nano Energy.

[15] Li C., Sleppy J., Dhasmana N., Soliman M., Tetard L., Thomas J. (2016). A PCBM-assisted perovskite growth process to fabricate
high efficiency semitransparent solar cells. J. Mater. Chem. A.

[16] Priyanka E., Muchahary D. (2024). Performance improvement of perovskite/CIGS tandem solar
cell using barium stannate charge transport layer and achieving PCE
of 39% numerically. Sol. Energy.

[17] Cao J., You P., Tang G., Yan F. (2023). Two-dimensional materials for boosting
the performance of perovskite solar cells: Fundamentals, materials
and devices. Mater. Sci. Eng., R.

[18] Novoselov K. S., Mishchenko A., Carvalho A., Castro Neto A. H. (2016). 2D materials
and van der Waals heterostructures. Science.

[19] Bonaccorso F., Sun Z., Hasan T., Ferrari A. C. (2010). Graphene photonics and optoelectronics. Nat. Photonics.

[20] Novoselov K. S., Geim A. K., Morozov S. V. (2004). Electric
Field Effect
in Atomically Thin Carbon Films. Science.

[21] Szeleszczuk, Mądra-Gackowska Ł., Gackowski M. (2025). Optoelectronic
and thermoelectric performance of cubic
X2YInO6 (X = Ba, Sr; Y = Nb, V) double perovskites: A first-principles
approach. Phys. B.

[22] Mądra-Gackowska K., Gackowski M., Szeleszczuk (2026). Density functional
theory insights into A2BBiCl6 (A
= Cs, K; B = Ag, Au) halide double perovskites for next-generation
photovoltaics. Phys. B.

[23] Szeleszczuk Ł., Mądra-Gackowska K., Hacholli V. B., Gackowski M. (2026). Designing
efficient energy materials: Optoelectronic and thermoelectric perspectives
of X2YBiCl6 (X = Cs, Na; Y = Ag, Au) double perovskites. J. Phys. Chem. Solids.

[24] Pereira V. S., Leal L. A., Ribeiro L. A., Blawid S. (2019). Inferring
changes in -stack mobility induced by aging from vibronic transitions
in poly­(3-hexylthiophene-2, 5-diyl) films. Synth.
Met..

[25] Kastinen T., da Silva Filho D. A., Paunonen L., Linares M., Ribeiro L. A., Cramariuc O., Hukka T. I. (2019). Electronic couplings
and rates of excited state charge transfer processes at poly­(thiophene-co-quinoxaline)–PC71BM
interfaces: two- versus multi-state treatments. Phys. Chem. Chem. Phys..

[26] Wang R., Mujahid M., Duan Y., Wang Z., Xue J., Yang Y. (2019). A Review of Perovskites
Solar Cell Stability. Adv. Funct. Mater..

[27] Potlog T., Dumitriu P., Dobromir M., Manole A., Luca D. (2015). Nb-doped TiO2
thin films for photovoltaic applications. Mater.
Des..

[28] Liang X., Ren X., Yang S., Liu L., Xiong W., Cheng L., Ma T., Liu A. (2021). Theoretical study of the influence of doped niobium
on the electronic properties of CsPbBr_3_. Nanoscale Adv..

[29] Ghorbani E., Barragan-Yani D., Albe K. (2020). Towards intermediate-band photovoltaic
absorbers: theoretical insights on the incorporation of Ti and Nb
in In2S3. npj Comput. Mater..

[30] Fernandes S. L., Simão G., Affonço L. J., Dias H., Longo E., Frederico C. (2019). Exploring
the Properties of Niobium Oxide Films for
Electron Transport Layers in Perovskite Solar Cells. Front. Chem..

[31] Giannozzi P., Andreussi O., Brumme T., Bunau O., Nardelli M. B., Calandra M., Car R., Cavazzoni C., Ceresoli D., Cococcioni M. (2017). Advanced capabilities
for materials modelling with Quantum ESPRESSO. J. Phys.: Condens. Matter.

[32] Giannozzi P., Baroni S., Bonini N., Calandra M., Car R., Cavazzoni C., Ceresoli D., Chiarotti G. L., Cococcioni M., Dabo I. (2009). QUANTUM ESPRESSO: A
modular and open-source software project for quantumsimulations of
materials. J. Phys.: Condens. Matter.

[33] Giannozzi P., Baseggio O., Bonfà P., Brunato D., Car R., Carnimeo I., Cavazzoni C., De Gironcoli S., Delugas P., Ruffino F. F. (2020). Quantum
ESPRESSO toward
the exascale. J. Chem. Phys..

[34] Perdew J. P., Burke K., Wang Y. (1996). Generalized gradient approximation
for the exchange-correlation hole of a many-electron system. Phys. Rev. B.

[35] Van
Setten M. J., Giantomassi M., Bousquet E., Verstraete M. J., Hamann D. R., Gonze X., Rignanese G.-M. (2018). The PseudoDojo:
Training and grading a 85 element optimized norm-conserving pseudopotential
table. Comput. Phys. Commun..

[36] Canning A. (2001). Thomas-Fermi
charge mixing for obtaining self-consistency in density. Phys. Rev. B.

[37] Cohen A. J., Mori-Sánchez P., Yang W. (2008). Fractional charge perspective on
the band gap in density-functional theory. Phys.
Rev. B.

[38] Crowley J. M., Tahir-Kheli J., Goddard W. A. (2016). Resolution of the
Band Gap Prediction
Problem for Materials Design. J. Phys. Chem.
Lett..

[39] Heyd J., Scuseria G. E. (2004). Efficient hybrid density functional calculations in
solids: Assessment of the Heyd-Scuseria-Ernzerhof screened Coulomb
hybrid functional. J. Chem. Phys..

[40] Hummer K., Harl J., Kresse G. (2009). Heyd-Scuseria-Ernzerhof
hybrid functional
for calculating the lattice dynamics of semiconductors. Phys. Rev. B.

[41] Lin L. (2016). Adaptively
compressed exchange operator. J. Chem. Theory
Comput..

[42] Gygi F., Baldereschi A. (1986). Self-consistent
Hartree-Fock and screened-exchange
calculations in solids: Application to silicon. Phys. Rev. B.

[43] Baroni S., De Gironcoli S., Corso A. D., Giannozzi P. (2001). Phonons and
related crystal properties from density-functional perturbation theory. Rev. Mod. Phys..

[44] di Meo, R. ; Corso, A. D. ; Giannozzi, P. ; Cozzini, S. Calculation of Phonon Dispersions on the Grid Using Quantum ESPRESSO. 2009.

[45] Golesorkhtabar R., Pavone P., Spitaler J., Puschnig P., Draxl C. (2013). ElaStic: A
tool for calculating second-order elastic constants from first principles. Comput. Phys. Commun..

[46] Dias A. C., Silveira J. F., Qu F. (2023). WanTiBEXOS: AWannier
based Tight
Binding code for electronic band structure, excitonic and optoelectronic
properties of solids. Comput. Phys. Commun..

[47] Mostofi A. A., Yates J. R., Lee Y.-S., Souza I., Vanderbilt D., Marzari N. (2008). wannier90: A tool for
obtaining maximally-localised
Wannier functions. Comput. Phys. Commun..

[48] Rozzi C. A., Varsano D., Marini A., Gross E. K. U., Rubio A. (2006). Exact Coulomb
cutoff technique for supercell calculations. Phys. Rev. B.

[49] Pizzi G., Volja D., Kozinsky B., Fornari M., Marzari N. (2014). BoltzWann:
A code for the evaluation of thermoelectric and electronic transport
properties with a maximally-localized Wannier functions basis. Comput. Phys. Commun..

[50] Rezaei S. E., Zebarjadi M., Esfarjani K. (2022). Calculation
of thermomagnetic properties
using first-principles density functional theory. Comput. Mater. Sci..

[51] Marzari N., Mostofi A. A., Yates J. R., Souza I., Vanderbilt D. (2012). Maximally
localized Wannier functions: Theory and applications. Rev. Mod. Phys..

[52] Kohn W. (1959). Analytic properties
of Bloch waves and Wannier functions. Phys.
Rev..

[53] Haastrup S., Strange M., Pandey M., Deilmann T., Schmidt P. S., Hinsche N. F., Gjerding M. N., Torelli D., Larsen P. M., Riis-Jensen A. C. (2018). The Computational 2D Materials Database: high-throughput
modeling and discovery of atomically thin crystals. 2D Mater..

[54] Gjerding M. N., Taghizadeh A., Rasmussen A., Ali S., Bertoldo F., Deilmann T., Knøsgaard N. R., Kruse M., Larsen A. H., Manti S. (2021). Recent
progress of the computational 2D materials database
(C2DB). 2D Mater..

[55] Kittel, C. ; McEuen, P. Introduction to Solid State Physics; John Wiley & Sons, 2018 10.1107/S0365110X54000448.

[56] Wang Y., Tian W., Zhang H., Wang Y. (2021). Nb 2 N monolayer
as
a promising anode material for Li/Na/K/Ca-ion batteries: a DFT calculation. Phys. Chem. Chem. Phys..

[57] Wang J., Bai L., Zhao X., Gao H., Niu L. (2022). A DFT prediction of
two-dimensional MB 3 (M= V, Nb, and Ta) monolayers as excellent anode
materials for lithium-ion batteries. RSC Adv..

[58] Tahir J. A., Ibeh G. J., Shuaibu A. (2024). First Principle
Study of Structural,
Electronic, Mechanical and Optical Properties of Bulk Niobium Dichalcogenide
NbX2 (X= S, SE) within a Visible Phonon Energy Range. Physics Access.

[59] Sun Y., Shen H.-X., Qiu Y., Fu H.-X., Duan M.-Y., Cheng C. (2024). Highly anisotropic
thermoelectric properties of the monolayer NbOX2
(X= Cl, Br, I) via first-principles calculations. Comput. Mater. Sci..

[60] Santos W., Pereira M., Frazão N., Moreira E., Azevedo D. (2024). 1T’-RuWTe2
hybrid monolayer as a novel magnetic material: A first principles
study. Mater. Today Commun..

[61] Barbosa L., Santos W., Moreira E., Azevedo D. (2025). Properties of OsIX-type
Janus monolayer with (X= Cl and Br), a comparative DFT study. Comput. Condens. Matter.

[62] Moujaes E. A., Diery W. (2019). Thermoelectric properties of 1 T
monolayer pristine and Janus Pd
dichalcogenides. J. Phys.: Condens. Matter.

[63] Diery W., Alharbi O. K., Moujaes E. A. (2025). From electronic
transport to thermoelectric
properties: The distinctive characteristics of non-Janus 1T-PtSSe
monolayers. Mater. Today Commun..

[64] Born M. (1940). On the stability
of crystal lattices. I. Math. Proc. Cambridge
Philos. Soc..

[65] Tromer R. M., Júnior L. R., Galvão D. S., Dias A. C., Moujaes E. A. (2024). On the
mechanical, thermoelectric, and excitonic properties of Tetragraphene
monolayer. Mater. Today Commun..

[66] Dias A. C., Bragança H., de Mendonça J.
P. A., Da Silva J. L. F. (2021). Excitonic
Effects on Two-Dimensional Transition-Metal Dichalcogenide Monolayers:
Impact on Solar Cell Efficiency. ACS. Appl.
Energy Mater..

[67] Dias A. C., Cornélio C. D. A., Piotrowski M. J., Júnior L. A. R., de Oliveira Bastos C.
M., Rêgo C. R. C., Guedes-Sobrinho D. (2024). Can 2D Carbon Allotropes Be Used
as Photovoltaic Absorbers in Solar Harvesting Devices?. ACS Appl. Energy Mater..

[68] Aparicio-Huacarpuma B. D., Pereira M. L., Piotrowski M. J., Rêgo C. R. C., Guedes-Sobrinho D., Ribeiro L. A., Dias A. C. (2025). Enhanced
solar harvesting efficiency in nanostructured MXene monolayers based
on scandium and yttrium. Nanoscale.

[69] Shockley W., Queisser H. J. (1961). Detailed Balance
Limit of Efficiency of p-n Junction
Solar Cells. J. Appl. Phys..

[70] Santos W. O., W S A., Carla F., Raimundo E., Rodrigues A. M., Frazão N. F., A, do D. (2024). Understanding
the optoelectronic and vibrational properties
of MB_4_O_7_, using density functional theory, where
M = Yb or Ce. Opt. Mater..

[71] Yu L., Zunger A. (2012). Identification
of Potential Photovoltaic Absorbers
Based on First-Principles Spectroscopic Screening of Materials. Phys. Rev. Lett..

[72] ASTM-G173–03 Standard Tables for Reference Solar Spectral Irradiances: Direct Normal and Hemispherical on 37 degree Tilted Surface, ASTM International, West Conshohocken, PA (2012). 2012 10.1520/g0173-03r20.

[73] Bernardi M., Palummo M., Grossman J. C. (2013). Extraordinary
Sunlight Absorption
and One Nanometer Thick Photovoltaics Using Two-Dimensional Monolayer
Materials. Nano Lett..

[74] Dias A. C., Lima M. P., Da Silva J. L. F. (2021). Role
of Structural Phases and Octahedra
Distortions in the Optoelectronic and Excitonic Properties of CsGeX3
(X = Cl, Br, I) Perovskites. J. Phys. Chem.
C.

[75] Silveira J. F. R. V., Besse R., Dias A. C., Caturello N. A. M. S., Da Silva J. L. F. (2022). Tailoring Excitonic and Optoelectronic
Properties of Transition Metal Dichalcogenide Bilayers. J. Phys. Chem. C.

[76] Moujaes E. A., Dias A. C. (2023). On the excitonic effects of the 1T
and 1OT phases of
PdS2, PdSe2, and PdSSe monolayers. J. Phys.
Chem. Solids.

[77] Jariwala D., Davoyan A. R., Wong J., Atwater H. A. (2017). Van der Waals Materials
for Atomically-Thin Photovoltaics: Promise and Outlook. ACS Photonics.

[78] Neamen, D. A. ; Biswas, D. Semiconductor Physics and Devices; McGraw-Hill Higher Education: New York, 2011 10.1002/0470068329.

[79] Musiienko A., Yang F., Gries T. W., Frasca C., Friedrich D., Al-Ashouri A., Sağlamkaya E., Lang F., Kojda D., Huang Y.-T. (2024). Resolving electron and hole transport properties
in semiconductor materials by constant light-induced magneto transport. Nat. Commun..

[80] Darwish M. N., Lentz J. L., Pinto M. R., Zeitzoff P. M., Krutsick T. J., Vuong H. H. (1997). An improved electron and hole mobility
model for general
purpose device simulation. IEEE Trans. Electron
Devices.

[81] Choi H. H., Cho K., Frisbie C. D., Sirringhaus H., Podzorov V. (2018). Critical assessment
of charge mobility extraction in FETs. Nat.
Mater..

[82] Shuai, Z. ; Wang, L. ; Song, C. Deformation potential theory. In Theory of Charge Transport in Carbon Electronic Materials; Springer, 2012 ; pp 67–88 10.1007/978-3-642-25076-7.

[83] Bardeen J., Shockley W. (1950). Deformation potentials
and mobilities in non-polar
crystals. Phys. Rev..

[84] Raval D., Moujaes E. A., Gupta S. K., Gajjar P. (2022). Thermoelectric properties
and the effect of biaxial strain and external electric fields on the
electronics of novel 2D Lace-like O-Pd2Q3 (Q = S, Se) monolayers. Surf. Interfaces.

[85] Raval D., Moujaes E. A., Gupta S. K., Gajjar P. (2024). Novel penta-GeC5
nanosheet
as potential candidate for efficient thermoelectric application: A
DFT approach. J. Phys. Chem. Solids.

[86] Barot J. B., Moujaes E. A., Gupta S. K., Gajjar P. (2025). Unveiling the optical
and thermoelectric properties of topological AsO and SbO monolayers
from the first principles study. Mater. Sci.
Eng. B.

[87] Harris, S. An Introduction to the Theory of the Boltzmann Equation; Courier Corporation, 2004.

[88] Jones, W. ; March, N. H. Theoretical solid state physics; Courier Corporation, 1985; Vol. 35.

[89] Lee M.-S., Mahanti S. D. (2012). Validity of the rigid band approximation in the study
of the thermopower of narrow band gap semiconductors. Phys. Rev. B.

[90] Takagiwa Y., Pei Y., Pomrehn G., Jeffrey Snyder G. (2013). Validity of rigid band approximation
of PbTe thermoelectric materials. Apl Mater..

[91] Morelli, D. T. ; Slack, G. A. High Thermal Conductivity Materials; Springer, 2006 ; pp 37–68 10.1007/0-387-25100-6_2.

[92] Slack G. A. (1965). Thermal
conductivity of elements with complex lattices: B, P, S. Phys. Rev..

[93] Qin G., Huang A., Liu Y., Wang H., Qin Z., Jiang X., Zhao J., Hu J., Hu M. (2022). High-throughput
computational evaluation of lattice thermal conductivity using an
optimized Slack model. Mater. Adv..

[94] Anderson O. L. (1963). A simplified
method for calculating the Debye temperature from elastic constants. J. Phys. Chem. Solids.

[95] Sanditov D. S., Mantatov V., Darmaev M., Sanditov B. (2009). On the Grüneisen
parameter for crystals and glasses. Tech. Phys..

[96] Julian C. L. (1965). Theory
of heat conduction in rare-gas crystals. Phys.
Rev..

[97] Peng B., Zhang H., Shao H., Xu Y., Zhang X., Zhu H. (2016). Thermal conductivity of monolayer
MoS_2_, MoSe_2_, and WS_2_: interplay of
mass effect, interatomic bonding
and anharmonicity. RSC Adv..

